# Promising early-career (bio)analytical researchers in 2026

**DOI:** 10.1007/s00216-025-06216-0

**Published:** 2025-11-22

**Authors:** Antje J. Baeumner, Soledad Cárdenas, Alberto Cavazzini

**Affiliations:** 1 Institute of Analytical Chemistry, Chemo- and Biosensors, University of Regensburg, Universitätsstraße 31, 93053 Regensburg, Germany; 2 Affordable and Sustainable Sample Preparation (AS 2P) Research Group, Departamento de Química Analítica, Instituto Químico para la Energía y el Medioambiente IQUEMA, Universidad de Córdoba, Campus de Rabanales, Edificio Marie Curie, 14071 Córdoba, Spain; 3 Department of Chemical, Pharmaceutical and Agricultural Sciences, University of Ferrara, via L. Borsari 46, 44121 Ferrara, Italy

We proudly present the second topical collection devoted to early-career researchers in ABC. Just like in 2021, we asked a selection of our fellow senior colleagues across the globe to nominate their brightest for this special collection and received very many wonderful nominations. An early-career researcher (ECR) is considered generally to be within 3 years before and after the award of their PhD before becoming a fully independent researcher. The editors of ABC decided to provide these researchers a public platform of recognition. This may assist all of them in this important career stage and simultaneously provides our scientific community with a broad spectrum of manuscripts describing relevant, inspiring, and exciting analytical and bioanalytical chemistry research.

The over 26 scientific researchers presented in this topical collection cover a broad range of analytical and bioanalytical chemistry in original research manuscripts and critical reviews. Research leads us through sample preparation strategies of extraction, separation, and chromatography to detection via MS, electrochemical, chemiluminescent, photothermal, Raman imaging, SERS, and fluorescent approaches. Since no analytical technology can be separated from the specifics of analyte and sample matrix, the application areas of the highlighted manuscripts are also of great interest. Clinical analyses focus, e.g., on gender-specific biomarkers for disease severity, on biopharmaceutical detection, RNA–lipid nanoparticles for therapy, point-of-care, exhaled breath analysis, forensics, and cancer detection. Environmental and food-related applications look, e.g., into pesticide, PFAS, wastewater, and heavy metal quantification. Novel materials and methodologies are utilized in these new assays, principles, and methods including nanomaterials, MOFs, application of nanozymes, CRISPR/Cas, and imprinted proteins. The variety of topics included in this collection highlights the importance of (bio)analytical chemistry in providing reliable solutions to information requirements in clinical, environmental, agrifood, and even instrumental development fields. These methodologies also consider sustainability by using miniaturized/automated sample preparation approaches, green solvents, and on-site analysis, among others.

All manuscripts submitted went through the regular peer-review process prior to acceptance in this issue. We all know how important this independent and anonymous process is to ensure the highest quality of our manuscripts. We also all know how challenging it can be to address differing views made by reviewers, how we sometimes engage in an in-depth scientific discussion during this process. Thus, this peer-review process, with its balanced combination of positive feedback and constructive criticism, has not only improved the quality of the submitted manuscripts but also helped ECRs articulate the innovative aspects of their research more effectively. A rigorous and serious peer revision is essential not only to uphold the scientific quality and ethical standards but also to build researchers’ confidence and professionalism. Indeed, while conducting research is fundamental, effectively communicating and disseminating the findings is equally crucial for advancing knowledge and establishing one’s scientific contributions. This dual focus empowers the new generation of researchers to face the challenges of their profession with integrity, dedication, and the necessary skills to share their work broadly. Like in the first edition, we expect this topical collection will help early-career researchers to be noticed in this highly competitive scholarly world and will help them to carve out a successful career path for themselves in the years to come. Thanks to the participants all over the world for their excellent contributions, and we wish you happy reading!

